# Cytokine response to resistance exercise in children with excess adiposity and Prader‐Willi syndrome

**DOI:** 10.14814/phy2.70327

**Published:** 2025-04-17

**Authors:** Vincent Vuong, Andrea M. Haqq, Daniela A. Rubin

**Affiliations:** ^1^ Department of Kinesiology California State University Fullerton Fullerton California USA; ^2^ Department of Pediatrics University of Alberta Edmonton Alberta Canada; ^3^ Department of Agricultural, Food & Nutritional Science University of Alberta Edmonton Alberta Canada

**Keywords:** children, interleunkin‐6, irisin, resistance exercise, TNF‐α

## Abstract

Interleukin‐6 (IL‐6), tumor necrosis factor alpha (TNF‐α), and irisin (cytokines) are affected by excess body fat (obesity), skeletal muscle, and resistance exercise (RE). The purpose of this study is to determine whether Prader‐Willi Syndrome (PWS), a genetic cause for obesity (OB), or non‐syndromic OB influences these cytokine responses to RE. Nine children with PWS (11.4 ± 3.3 years, 45.6 ± 5.2% BF), 11 children without OB (9.2 ± 1.4 years, 18.6 ± 5.0% BF), and 12 children with OB (9.6 ± 1.3 years, 40.4 ± 5.4% BF) participated. Children stepped onto an elevated platform wearing a weighted vest for 6 sets of 10 repetitions per leg separated by 1 min of rest. Blood samples were obtained before exercise (pre), immediately post (IP), and during recovery (+15 and +60 min). There were no group‐by‐time interactions for any cytokine; and neither time nor group effects for TNF‐α or irisin (*p* ≥ 0.378). For IL‐6, 60+ was higher than pre, IP, and +15 (*p* < 0.001). Children with PWS and OB had increased IL‐6 than children without OB (*p* ≤ 0.038). Neither PWS nor OB affected IL‐6, TNF‐α or irisin responses to RE. However, excess body fat was associated with higher IL‐6 concentrations.

## 1 INTRODUCTION

Obesity is defined as an abnormal or excess accumulation of fat mass (Apovian, 2016; Sanyaolu et al., 2019). Children ages 2–19 are considered to present with obesity if their body mass index (BMI) falls within the 95th BMI percentile for their respective sex and age (Centers for Disease Control and Prevention, 2022). In the United States, the Centers for Disease Control and Prevention have recently reported obesity affected 19.7% of children between 2017 and 2020 (Centers for Disease Control and Prevention, 2022). Obesity in childhood is of concern as it is associated with low‐grade systemic inflammation, type 2 diabetes, cardiovascular disease, respiratory conditions, and poor health‐related quality of life (Calcaterra et al., 2020; Sanyaolu et al., 2019).

Prader‐Willi syndrome (PWS) is a rare genetic disorder that develops from an error in genomic imprinting in the expression of the paternal chromosome 15q11‐q13 region (Butler et al., 2019). PWS results in congenital excess of body fat, hormonal alterations, and lack of satiety. Insufficient growth hormone contributes to short stature, excess adiposity, and decreased lean mass (Cassidy et al., 2012). Growth hormone replacement therapy (GHRT) in childhood leads to normalized height, increased lean mass, and decreased fat (Gondoni et al., 2008). However, even with GHRT, lean mass and fat mass in individuals with PWS still remain outside of normal levels, with increased fat and decreased lean mass (Gondoni et al., 2008; Lloret‐Linares et al., 2013).

Interleukin‐6 (IL‐6) and tumor necrotic factor‐alpha (TNF‐α) are two cytokines associated with obesity and risk factors for adverse health (Rubin & Hackney, 2010). IL‐6 and TNF‐α are produced and released from the adipose tissue, T cells, monocytes, and macrophages (Arroyo et al., 2020; Eaton et al., 2018). However, IL‐6 is also produced and released by the skeletal muscle when the muscle contracts (Steensberg et al., 2002). IL‐6 increases glucose oxidation and lipolysis, decreasing insulin resistance (Pedersen & Febbraio, 2007) but can also lead to insulin resistance (Klover et al., 2003; Lin et al., 2023). TNF‐α is a pro‐inflammatory cytokine involved in systemic inflammation, triggering acute phase pro‐inflammatory responses; it is associated with cardiometabolic disease, including insulin resistance (Nieto‐Vazquez et al., 2008; Rubin et al., 2008; Rubin & Hackney, 2010), muscle loss, and lower muscular strength (McMahon et al., 2019). Irisin is an anti‐inflammatory cytokine produced and released by myocytes during muscular contraction (Huh, Mougios, et al., 2014; Lee et al., 2015; Li et al., 2021). It is responsible for various physiological actions, the main ones being the conversion of white adipose tissues into brown adipose tissue, activating thermogenesis (Bostrom et al., 2012), glucose uptake by the muscle (Lee et al., 2015), and reducing insulin resistance (Yano et al., 2020). In addition, irisin contributes to muscle growth and differentiation by stimulating the expression of myoblasts by activating downstream extracellular signal‐regulated kinase 1/2 pathways and IL‐6 pathways (Huh, Dincer, et al., 2014).

Acute resistance exercise (RE) induces IL‐6 and irisin secretion immediately post exercise and during recovery in prepubertal boys and adult men with and without metabolic syndrome (Huh et al., 2015; Jansson et al., 2025; Tsuchiya et al., 2015). Elevated TNF‐α concentrations have been demonstrated in adult men in response to prolonged endurance exercise in the heat (Arroyo et al., 2020), in adolescents in response to high intensity swimming (Sanderson et al., 2020) and in children after a resistance training session (Jansson et al., 2025). However, other studies found RE does not appear to lead to acute increases in TNF‐α in young and middle‐aged males (Arroyo et al., 2017; Libardi et al., 2011). The response of these cytokines to acute exercise is of interest as some of the chronic adaptations resulting from RE in metabolism and the skeletal muscle appear to be mediated by these cytokines (Docherty et al., 2022; Liu et al., 2022).

Previous studies have shown the importance of RE training in improving endocrine function in children with excess adiposity (Bell et al., 2007; Garcia‐Hermoso et al., 2017). RE has also been shown to reduce body fat in children with obesity (Dias et al., 2015). In children and adolescents, aerobic plus resistance exercise training reduces low‐grade systemic inflammation (Damaso et al., 2014; Wong et al., 2018). In children, two studies to date evaluated the role of acute RE affecting the concentrations of these cytokines (Blizzard LeBlanc et al., 2017; Jansson et al., 2025). As these cytokines are related to metabolism, understanding these responses is of interest. As excess fat or reduced lean mass may influence the responses, children with PWS may present altogether a different response. Therefore, this study examined IL‐6, TNF‐α, and irisin responses to an acute bout of resistance exercise in children with PWS and compare their responses to children with and without obesity. We hypothesize that IL‐6 and irisin will increase in all groups in response to exercise while TNF‐α may not change. Obesity independently of the genetic condition will exacerbate the levels of all cytokines.

## 2 MATERIALS AND METHODS

### 2.1 Participants

The study was approved by the Institutional Review Boards of California State University Fullerton, the Children's Hospital of Orange County, and the Human Research Protection Office of the United States Army Medical Research and Materiel Command. All participants and parents signed informed assent and consent forms. A screening questionnaire was completed by the parents of the participants, and a medical examination was performed on the participants with PWS to identify contraindications to participation in the study. Children with PWS were required to have a genetic confirmatory diagnosis for participation. Diagnosis included: uniparental deletion (*n* = 8) and one unknown. Current medications for children with PWS included GH (*n* = 5), diabetes medication (*n* = 1), Albuterol (*n* = 1), inhaled steroids (*n* = 2) antidepressants (*n* = 1), testosterone (*n* = 1), and coenzyme Q_10_ (*n* = 1). Due to the exercise protocol including heavy loading on the spine, orthopedic problems in PWS were considered before participation in the study. Participants without PWS were excluded if they exhibited insulin resistance, type 2 diabetes mellitus, or other metabolic diseases. Participants with confirmed pregnancy or inability to participate in a vigorous physical activity were also excluded from participation. Participants without PWS included participants without obesity (body fat percentage <85th percentile for age and sex) and participants with obesity (body fat percentage >95th percentile for age and sex) (McCarthy et al., 2006).

### 2.2 Exercise trial

Participants were asked to abstain from exercise the day before the exercise test and to consume their habitual diet, which was verbally verified upon arrival to the Exercise Physiology Laboratory at California State University Fullerton or the Endocrine Department at the Children's Hospital of Orange County. Participants consumed a standardized breakfast 2 h before reporting to the testing sites. The breakfast did not contain caffeine and consisted of a nutritional bar and reduced‐sugar apple sauce, and water ad libitum (260 kcal/1088 kJ) (7 g of fat [21.5%], 37 g of carbohydrates [57%], and 14 g of protein [21.5%]). Upon arrival, children were seated, and an indwelling catheter was placed in an antecubital or dorsum‐of‐hand vein. A resting blood sample was obtained 30 min after catheter insertion.

Afterwards, participants were fitted with a heart rate (HR) monitor and completed a 5‐min cycling or walking warm‐up to elevate HR above 120 bpm. Immediately following, children completed the resistance protocol which consisted of 6 sets of 10 repetitions per leg of a step‐up exercise onto a platform while wearing a weighted vest; in between sets, participants completed a seated rest for 1‐min. A repetition for the right leg was counted as up right, up left, down right, down left; a repetition for the left leg was counted as up left, up right, down left, down right. The amount of relative work was standardized for children classified as lean or with obesity by varying the load of the vest (50% of lean body mass) and height of the platform (20% of stature). Regardless of stature, all children with PWS used a platform height of 23.0 cm, due to the morphological and balance constraints. The formula: vest load (kg) = (20% of stature [cm] × 50% of lean body mass [kg])/23.0 cm was used to calculate the vest load for each child with PWS to provide an equivalent total work in all three groups. HR was continuously monitored using telemetry (Polar USA, Lake Success, NY, USA) and both HR and ratings of perceived exertion (RPE) were obtained at the end of each set (Robertson et al., 2005). Blood samples were obtained before exercise (PRE), immediately post exercise (IP), and during recovery from exercise (+15 and +60 min) while the children were in a seated position.

### 2.3 Hormone and metabolite measurement

Approximately 5 mL of blood was placed in chilled tubes pretreated with EDTA (BD Diagnostics, Franklin Lakes, NJ, USA). The samples were centrifuged at 4°C for 15 min at 3000 rpm. The resulting plasma was aliquoted and frozen at −80°C until in‐house analyses were conducted at the Exercise Biochemistry Laboratory at California State University Fullerton (irisin) or at the Li Ka Shing Center at the University of Alberta (IL‐6 and TNF‐α). The samples were thawed only once and evaluated in duplicate during the same analytical run. Enzyme‐linked immunoassays were conducted to measure irisin (Catalog# EK‐067; Phoenix Pharmaceuticals, Burlingame, CA, USA). Cytometric Bead Array Assays were used to measure IL‐6 and TNF‐α (Catalog # 551811; BD Biosciences, San Jose, CA, USA). Intra‐assay coefficients of variation were 10.9% (IL‐6), 6.4% (TNF‐α), and 8.1% (irisin). Inter‐assay coefficients of variation were 12.4% (IL‐6), 16.6% (TNF‐α), and 10.0% (irisin). To accurately reflect the target tissues' actual exposure to cytokines, concentrations are reported as measured values not corrected for plasma volume shifts.

### 2.4 Statistical analyses

Frequencies, means, standard errors of the mean, and minimum and maximum values are presented as appropriate for all outcomes. Three (groups) by four (time points) repeated measures ANOVAs were conducted to determine differences in the cytokine responses to RE between the groups. Additionally, one‐way ANOVAs were used to compare participant characteristics, exercise responses, and AUC values for each cytokine between the groups. Post hoc pairwise comparisons (group comparisons and comparisons between time points) were done using the Bonferroni correction. Statistical significance was set at *p* < 0.050.

## 3 RESULTS

### 3.1 Exercise responses

Table 1 shows the participant characteristics and exercise responses to the resistance exercise protocol. There was no significant difference in stature or lean mass (kg) among the groups (*p* > 0.050). There were significant group differences in total body mass (kg), body fat (% and kg), lean mass (%), and fat mass to lean mass ratio (*p* ≤ 0.002 for all). Children with PWS and obesity had a greater body mass, body fat (% and kg) and fat mass to lean mass ratio than children without obesity (*p* < 0.014 for all). Children with PWS and with obesity had a lower lean mass % than those without obesity (*p* < 0.001). Children with PWS had a greater fat mass to lean mass ratio than children with obesity (*p =* 0.041). There was no significant difference in total body mass, lean mass, and fat mass between children with PWS and children with obesity (*p* > 0.050 for all). Children with PWS (*n* = 7) took longer to complete the test compared to children with obesity (*p* = 0.010) and without obesity (*p =* 0.020). There were no group differences for exercise mean HR, RPE, or exercise vest load (*p* > 0.050 for all).

**TABLE 1:** Participant characteristics and exercise responses to resistance exercise (RE). Values are displayed as mean ± SD.

	Children with PWS (*n* = 9)	Children with obesity (*n* = 12)	Children without obesity (*n* = 11)
Age (Years)	11.4 ± 3.3	9.1 ± 1.3	9.1 ± 1.4
Stature (cm)	144.4 ± 18.2	142.5 ± 7.6	139.9 ± 10.3
Body mass (kg)	53.5 ± 20.9	48.3 ± 10.5	31.2 ± 7.0
Body fat (%)	45.6 ± 5.2	40.4 ± 5.4	18.6 ± 5.0
Body fat (kg)	25.3 ± 11.4	20.4 ± 6.0	6.8 ± 2.7
Lean mass (%)	52.6 ± 4.9	57.7 ± 5.0	78.3 ± 5.0
Lean mass (kg)	27.5 ± 10.4	27.2 ± 5.2	24.0 ± 5.0
Fat mass: Lean mass ratio^a^, ^b^	0.9 ± 0.2	0.7 ± 0.2	0.3 ± 0.1
Test duration (min)^b^, ^c^	16.1 ± 3.8	12.3 ± 2.1	12.6 ± 1.6
Exercise responses			
Heart rate (bpm)	150.2 ± 18.9	163.6 ± 15.1	150.9 ± 18.7
RPE (1–10)	4.9 ± 2.4	5.7 ± 2.5	4.6 ± 1.7
Vest load (kg)	12.0 ± 2.5	13.7 ± 2.6	17.5 ± 8.3

*Note*: Participants with PWS *n* = 7 for test duration. Significance set at *p* < 0.05 for all analyses.

^a^ Marks significance between children with PWS and obesity versus children without obesity.

^b^ Marks significance between children with PWS versus children with obesity.

^c^ Marks significance between children with PWS versus children with obesity and without obesity.

### 3.2 Cytokine responses

**FIGURE 1:**
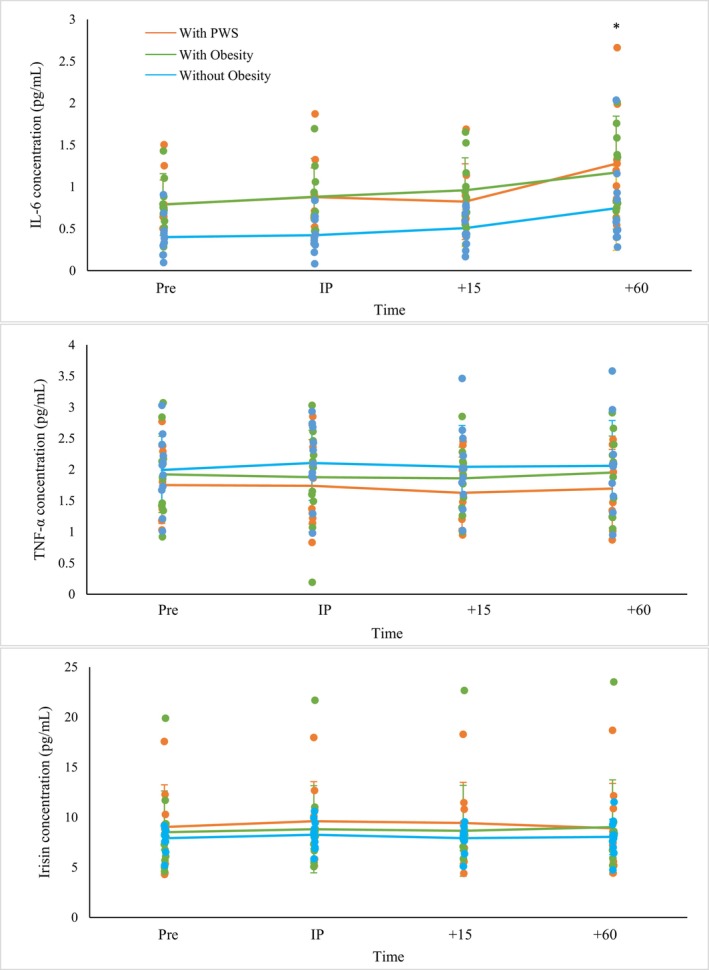
IL‐6, TNF‐α, and irisin responses to RE. IL‐6, TNF‐α, and irisin: No significant group by time interaction. IL‐6: +60 greater than all other time points, children with PWS (*n* = 9) or obesity (*n* = 12) greater than children without obesity (*n* = 11). TNF‐α and irisin: No time or group effects. Significance set at *p* < 0.050 for all analyses. *Indicates time point is different from other time points.

**FIGURE 2:**
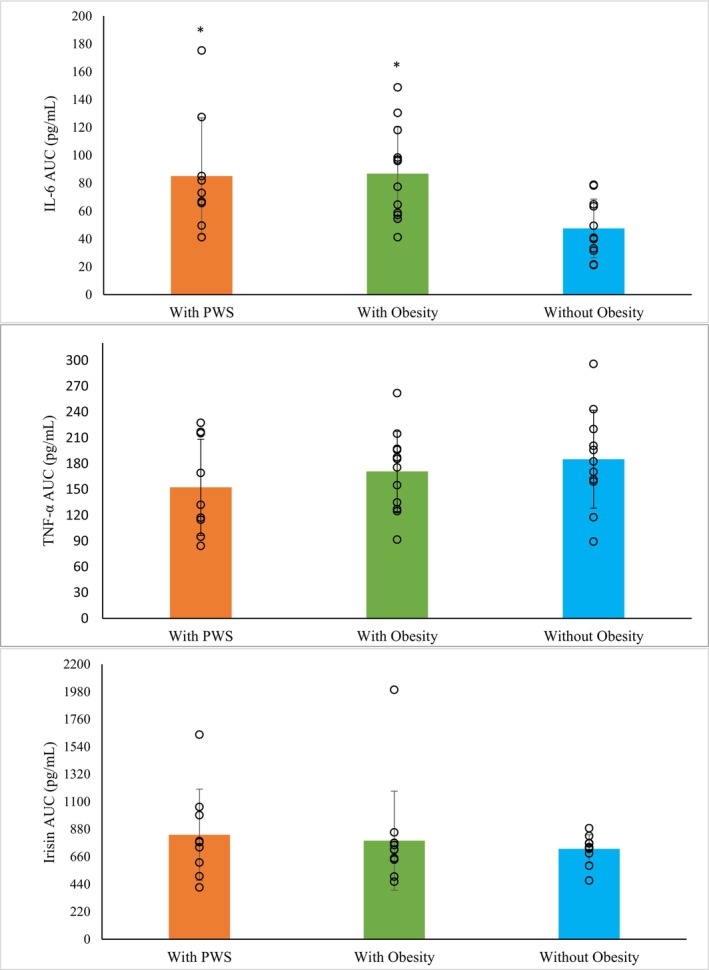
Area under the curve (AUC) for IL‐6, TNF‐α, and irisin. IL‐6: Children with PWS (*n* = 9) and with obesity (*n* = 12) show higher AUC than children without obesity (*n* = 11). TNF‐α and irisin: No significant group difference. Significance set at *p* < 0.050 for all analyses.

Figure 1 presents the cytokine responses to RE. There were no group by time interactions for IL‐6, TNF‐α or irisin (*p* ≥ 0.580 for all). A group effect (*p* = 0.011) and pairwise comparisons showed IL‐6 concentrations in children with PWS and children with obesity were greater than concentrations in children without obesity (0.94 ± 0.12 pg/mL and 0.95 ± 0.10 pg/mL vs. 0.52 ± 0.11 pg/mL; *p* = 0.038 and *p* = 0.020, respectively). A time effect (*p* < 0.001) and pairwise comparisons showed IL‐6 concentrations at +60 were greater than concentrations at PRE, IP and +15 (*p* < 0.001 for all). There were also no group differences for TNF‐α or irisin concentrations (*p* ≥ 0.430 for both). There were no differences over time for TNF‐α or irisin concentrations (*p* ≥ 0.289 for both). One‐way ANOVAs showed significant group differences for IL‐6 (*p* = 0.013) but no significant difference in AUC for TNF‐α (*p* = 0.402) or irisin (*p* = 0.731). Pairwise comparisons showed children with PWS (*p* = 0.047) and with obesity (*p* = 0.022) had a greater IL‐6 AUC compared to children without obesity (See Figure 2).

## 4 DISCUSSION

Thirty‐two children performed a resistance training protocol by performing step ups with a weighted vest to elicit a cytokine response 2 h after a carbohydrate‐rich meal. Neither PWS nor excess adiposity affected changes in the cytokine concentrations assessed immediately post‐exercise or during recovery from exercise. While IL‐6 concentrations increased during recovery from exercise, there were no significant increases in neither TNF‐α nor irisin to this exercise protocol. Children with excess body fat due to PWS or due to non‐syndromic obesity exhibited greater IL‐6 concentrations than those without excess body fat.

During exercise, IL‐6 is released from the contracting skeletal muscle and its release is dependent on the duration and intensity of the exercise and can be attenuated if consuming a diet high in carbohydrate or a carbohydrate‐containing solution during exercise (Gleeson & Bishop, 2000; Starkie et al., 2001). In the present study, increased IL‐6 concentrations were only observed at 60 min into recovery from exercise. If the participants had not been in a postprandial state, they might have demonstrated a higher concentration of IL‐6 at other time points (Gleeson & Bishop, 2000; Starkie et al., 2001). Children with and without obesity have shown increased IL‐6 concentrations immediately post‐exercise and an hour into recovery in response to moderate‐intensity aerobic exercise (Duran et al., 2015; Santos et al., 2019), high‐intensity interval training exercise (Cullen et al., 2016), and moderate‐intensity resistance exercise (Jansson et al., 2025). In adults, a RE protocol induced increased IL‐6 immediately post‐exercise in young men with and without obesity (Mitchell et al., 2011) and in young men with PWS (Hirsch et al., 2020). Hence, the present study confirms previous findings in children in terms of responses to exercise and in adults with PWS, which demonstrate that neither PWS nor excess body fat affect IL‐6 responses to exercise.

The excess adiposity exhibited by children with PWS and those without PWS but with non‐syndromic obesity likely is related to the greater overall concentrations of IL‐6. This is comparable to previous studies in children with excess adiposity (De Filippo et al., 2015; Duran et al., 2015; Rosa et al., 2011). Of note, the increased AUC for IL‐6 shown by those with PWS or obesity suggests the tissues are exposed to greater concentrations of this cytokine after exercise with potential good benefits such as lipolysis and muscle hypertrophy (Munoz‐Canoves et al., 2013; Steensberg et al., 2002).

Neither PWS nor excess adiposity or exercise influenced TNF‐α concentrations. Our findings are similar to other studies that failed to show an increase in TNF‐α in response to either vigorous cycling (Duran et al., 2015) or plyometrics (Kurgan et al., 2020). In contrast, two studies showed increased TNF‐α concentrations after vigorous intensity aerobic exercise in children with obesity (Rosa et al., 2011) and after a moderate intensity resistance training in prepubertal and pubertal boys (Jansson et al., 2025). TNF‐α has been shown to be released from macrophages and to respond to cellular damage as well as increase after strenuous prolonged exercise (Jang et al., 2021; Ostrowski et al., 1999). While it is tempting to speculate that IL‐6 may inhibit the response of TNF‐α to exercise (Schindler et al., 1990), as IL‐6 concentrations were not elevated until 60 min into recovery, this possibility is unlikely. The lack of TNF‐α response in our study may result from the exercise intensity not being strenuous enough or the shorter duration of exercise, as increased intensity and prolonged exercise have been shown to increase TNF‐α (Arroyo et al., 2020; Ostrowski et al., 1999). Likewise, similar to Duran et al. (2015) and Kurgan et al. (2020), we found no differences in TNF‐α concentrations based on levels of body fat.

Irisin is a cytokine released from the skeletal muscle and is directly influenced by exercise intensity (Huh et al., 2015; Loffler et al., 2015; Morelli et al., 2020). Studies in adults have shown an increase in irisin (~15%–20% change) following RE at 65% and 75%–80% of their one repetition maximum (Huh et al., 2015; Tsuchiya et al., 2015), and during 45–90 min of aerobic exercise at moderate to high intensity (Kraemer et al., 2014; Norheim et al., 2014) with increases observed immediately (Huh et al., 2015; Norheim et al., 2014) as well as 1 h after exercise (Tsuchiya et al., 2015). Our irisin findings are similar to studies in adults with and without obesity that found no change in irisin with high intensity exercise (Archundia‐Herrera et al., 2017; Fernandez‐del‐Valle et al., 2018). It is possible that the cumulative dose of the protocol (intensity and duration) in the present study might have not been enough to elicit a significant response in irisin. Across all groups, children reported RPE values of 5.2 ± 2.3 which translates to “getting more tired to tired” on the resistance exercise scale (Robertson et al., 2005) and had a heart rate of 155 ± 18.1 bpm, which is about 77.3% of their maximum heart rate (Tanaka et al., 2001) equating to vigorous exercise according to the American College of Sports Medicine aerobic exercise intensity guidelines (Zuhl, 2020). However, this protocol was selected because it could be conducted in a laboratory and a hospital setting accommodating children of different size and required little coordination and skill level, all necessary to conduct the study with children with PWS. It is also possible that repeated exposure to the same exercise stimulus is needed to trigger sufficient irisin release to detect acute increases in its concentration in the blood after exercise. A resistance training intervention in youth showed increased irisin release in response after the intervention (Blizzard LeBlanc et al., 2017).

In contrast to the present study, other studies have shown that irisin concentrations at rest are higher in children with obesity compared to those of normal weight (Catli et al., 2016; Nigro et al., 2017). Our groups, while they showed differences in fat mass and body fat percent, showed similar lean mass. Potentially, this lack of difference in lean mass (a surrogate for muscle mass) may explain the lack of differences among the groups. This is of interest, as usually children with PWS exhibit less muscle mass than children with non‐syndromic obesity (Lloret‐Linares et al., 2013).

### 4.1 Strengths and limitations

The present study is one of few that evaluated acute cytokine responses to RE in children with PWS compared to children with and without obesity. Despite the novelty of the results, some limitations need discussion. First, while the RE protocol was chosen because of its feasibility, it is likely the intensity was insufficient to trigger a cytokine response (Archundia‐Herrera et al., 2017; Cullen et al., 2016). The exercise protocol used in this study was an adapted version of a step‐up protocol previously used on adults (National Strength and Conditioning Association, 2008). Because the protocol included stepping up with 10 repetitions per leg, it included muscular endurance along with muscular strength and power. Second, the sample size was small and likely limited the statistical power to detect potential group (observed power TNF‐α = 0.185 and irisin = 0.094) or time (observed power TNF‐α = 0.101 and irisin = 0.204) differences. Some participants with PWS had been on GHRT (*n* = 5) while others have not (*n* = 4). Previous research has shown growth hormone can enhance IL‐6 and TNF‐α production (Uronen‐Hansson et al., 2003); hence the lack of homogeneity in GHRT in the group with PWS might have affected IL‐6 and TNF‐α findings. Third, cytokine responses were only analyzed immediately post exercise and during recovery and not during the exercise protocol. Fourth, there were no groups/conditions that addressed fasting or no exercise. This is a limitation due to the role of IL‐6 and TNF‐α in metabolic homeostasis which can be altered due to a fasted or fed state (Lin et al., 2023; Starkie et al., 2001) (Gleeson & Bishop, 2000) or exercise. Lastly, the peak of cytokine responses may be different between children with PWS and children with and without obesity. The present study used three post‐exercise time measurements and detected a peak in IL‐6 concentrations at 60 min with no significant difference in irisin and TNF‐α. Possibly, increases in these cytokines concentrations past 60 min into recovery from exercise could occur, but these increases would be cytokine and exercise mode dependent (Gleeson & Bishop, 2000; Ostrowski et al., 1999; Tsuchiya et al., 2015).

## 5 CONCLUSION

Neither PWS nor excess body fat affected cytokine responses to acute RE; however, excess body fat led to overall increased IL‐6 concentrations, confirming findings by others (Duran et al., 2015). Despite the possibility that this protocol might have been of insufficient intensity, the increased IL‐6 concentrations 1 h after exercise suggest a potential metabolic effect that can be gained through this type of exercise.

## FUNDING INFORMATION

Study funded by the US Army Medical Research and Materiel Command Contract W81XWH‐08‐1‐0025 (DAR).

## CONFLICT OF INTEREST STATEMENT

None of the authors have any competing interests to declare.

## ETHICS STATEMENT

This study was conducted in agreement with the ethical standards for responsible research involving human participants of the participating institutions and the Helsinski Declaration of 1975, as revised in revised 2008.

## Data Availability

Data are available upon request.
